# Clinical insights on the spasticity-plus syndrome in multiple sclerosis

**DOI:** 10.3389/fneur.2022.958665

**Published:** 2022-08-05

**Authors:** Kanza Alami Marrouni, Pierre Duquette

**Affiliations:** ^1^Department of Neurosciences, Faculty of Medicine, Université de Montréal, Montreal, QC, Canada; ^2^Centre de recherche du Centre hospitalier de l'Université de Montréal, Montreal, QC, Canada; ^3^Department of Neurology, Centre hospitalier de l'Université de Montréal, Montreal, QC, Canada

**Keywords:** multiple sclerosis, spasticity, spasticity-plus syndrome, cannabinoids, nabiximols, symptomatic therapy, neuroimaging

## Introduction

Multiple sclerosis (MS) is the most common chronic autoimmune neurodegenerative disease in young Caucasian adults (1). It is characterized by lesions of oligodendrocytes and myelin, in addition to neuronal and axonal injury, resulting in multiple neurological dysfunctions (1). Clinical symptoms include spasticity, pain, weakness, bladder, bowel, and sexual disturbances, sleep disorders, and more. According to a recent hypothesis, multiple symptoms associated with spasticity can be part of the same cluster in persons with MS (PwMS) (2). The spasticity-plus syndrome (SPS) was first introduced by Fernández et al. (2) who defined it as a cluster of the following MS symptoms: spasticity, spasms/cramps, pain, bladder dysfunction, sleep disorders, fatigue, and possibly tremor. Authors stated that these symptoms are mediated, in part, in the brainstem. Therefore, due to the wide distribution of cannabinoid receptors 1 and 2 in the central nervous system (CNS), particularly in the brainstem, they suggested that cannabinoids might play a higher role in the symptomatic treatment of MS than what is reported in the literature (2). Bruno et al. (3) detailed this hypothesized syndrome. In their paper, the SPS pathophysiology is explained by a greater axial resistance that hypersensitizes the demyelinated axons, leading to a conduction block and an ephaptic transmission. In this opinion article, we highlight the importance of validating the clustering of MS spasticity-related symptoms, as suggested in the SPS, by discussing the relevance in determining a reliable clinical assessment tool for the SPS, providing insights on neuroimaging, and presenting the clinical evidence on the symptomatic treatment with cannabinoids.

## Symptoms included in the spasticity-plus syndrome

**Figure 1:**
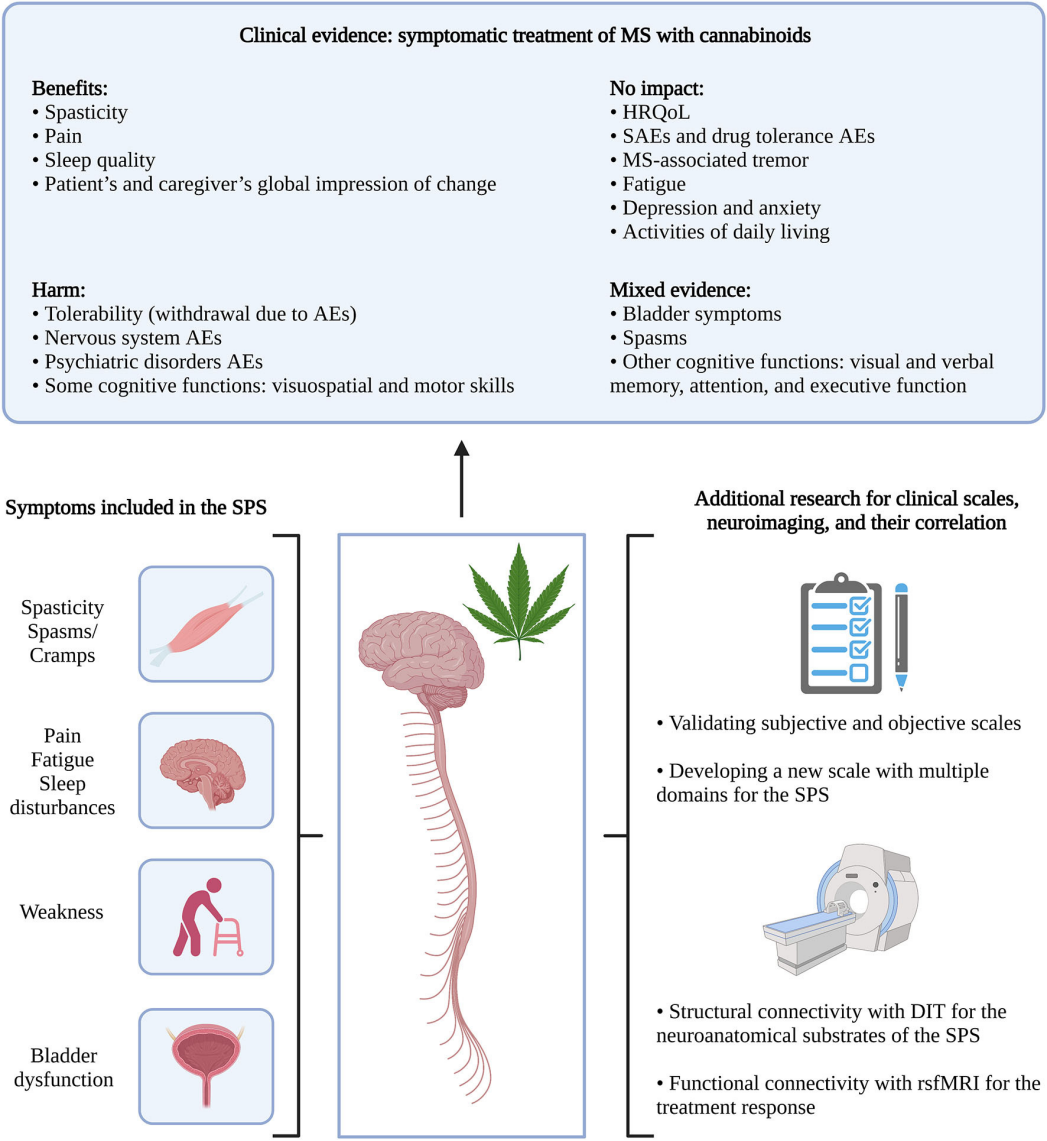
From the clinical evidence of MS symptomatic therapy with cannabinoids to the SPS. Clinical evidence was retrieved from systematic literature reviews by Filippini et al. (5) and Landrigan et al. (6). MS, spasticity; AEs, adverse events; HRQoL, health-related quality of life; SAEs, serious adverse events; SPS, spasticity-plus syndrome; DIT, diffusion imaging tractography; rsfMRI, resting-state functional magnetic resonance imaging. Created with BioRender.com.

The first hypothesis introduced by Fernández et al. (2) on the SPS included only spasticity-associated symptoms in MS and based the cluster on symptoms improving with cannabinoids, mainly nabiximols, a balanced combination of tetrahydrocannabinol and cannabidiol (2). When asked about clustering the suggested symptoms in one syndrome—excluding tremor—, a panel of MS expert neurologists in Spain (n = 55) was in favor of the SPS, on a 10-point scale (0 = totally disagree; 10 = totally agree), with a mean score of 8.16 ± 1.40 (4). Bruno et al. (3) later hypothesized the pathophysiology of the SPS, using the same symptoms in the SPS as originally described, except including weakness instead of tremor (Figure 1).

Multiple questionnaires are available to assess spasticity. Choosing the appropriate tool depends on the context (i.e., clinical research or clinical practice), if it is objective or subjective, and on its validity regarding a specific disease. Objective assessments report observable data by physical examination and/or measurable values by laboratory and diagnostic testing, such as the Modified Ashworth Scale for MS-associated spasticity (7). In contrast, subjective assessments are based on the patient's perspective, like the Numeric Rating Scale for spasticity in MS (8). Similarly, there are multiple assessment tools for the other symptoms. If the SPS model is validated, its evaluation could constitute a burden to both physicians, PwMS, and their caregivers, due to the overwhelming quantity of questionnaires, especially for severely impaired PwMS. Therefore, determining the most appropriate MS symptoms to include in this syndrome and the corresponding scales should be prioritized. Ideally, a single evaluation would address them all, such as an enlarged version of the MS Functional Composite score (MSFC), comprised of three tests assessing ambulation, dexterity, and cognitive impairment (9).

## Assessment of the spasticity-plus syndrome with imaging

According to Bruno et al. (3), both lateral and anterior corticospinal tracts are involved in MS, but with a greater sensitivity to demyelination for fibers in the former tract. Their axons have a small diameter which explains a higher axial resistance for the action potential propagation, leading to a conduction block. Demyelination enhances the axial resistance and favors an ephaptic transmission of the action potential. Therefore, authors divided the SPS symptoms in two pathophysiological categories. They suggested that conduction block would explain spasticity, weakness, fatigue, and urinary retention (3). In the case of spasticity, for example, nerve conduction block would facilitate the disinhibition of the dorsal reticulospinal tract, contributing to hyperreflexia, because this tract is affected by supraspinal lesions of the primary motor cortex (10). With a current shunt, damaged axons are forced to share their hyperexcitability with other axons (ephaptic transmission), which would explain spasms, pain, allodynia, and urinary urgency (3).

To validate this dual model of conduction block and ephaptic transmission in the symptomatic manifestations of MS, imaging techniques can be used. The structural connectivity network defines the association between different areas of the CNS, but cannot determine the direction, nor distinguish between excitatory or inhibitory connections (11, 12). It can be evaluated with diffusion imaging tractography (DIT), combining diffusion magnetic resonance imaging (MRI), such as diffusion tensor imaging, with the visual representation of nerve tracts by tractography or using tract atlases (12–14). This technique focuses more on tracing white matter connections but mapping disconnections between gray matter regions due to focal lesions is possible (12). In contrast, functional connectivity defines the correlation, in case of functional MRI (fMRI), or the coherence, in case of electro- or magnetoencephalogram (EEG/MEG) signals, between these nodes (11). fMRI suffers from limitations in temporal resolution and is seen with two methods, task-based fMRI, which varies according to the asked task to perform, and resting-state fMRI which avoids this task-based confounder (11, 12, 15). EEG is limited in spatial resolution, weakening the anatomic specificity, while MEG offers a high spatial and temporal accuracy (15). Lastly, effective connectivity, as inferred from a network model, helps determine the direction and the sign (excitatory or inhibitory) of neuronal interactions, but no *in vivo* imaging technique measures it directly (11, 12).

Multiple MS studies conducted neuroimaging to address the brain network connectivity of cognitive functions, pain, and fatigue, but few evaluated spasticity. A retrospective study reported three main regions with DIT for MS participants who developed spasticity: the genu or the posterior limb in the internal capsule, the rostral brainstem, and the callosal radiations cross interleaving with corticospinal tracts (16). Another small-sized study evaluated the impact of intermittent theta brain stimulation on MS participants with spasticity and showed the relevance of resting-state fMRI in highlighting the treatment effect on spasticity with a functional reorganization of the primary motor cortices, favoring connections of the contralateral primary motor cortex to other cerebral regions (17).

Most MS imaging studies focus on the brain to illustrate motor, sensitive, and cognitive impairment, even if some symptoms involve the spinal cord, such as spasticity. This is partly due to the challenging imaging of the spinal cord, since its axons have a small diameter and it is susceptible to motion artifacts (e.g., body fluid pulsation, breathing, and swallowing) (13, 14). Therefore, its imaging studies usually involve part of the spinal cord at high spatial resolution, then uses it as a proxy for its entirety (18). The Spinal Cord Toolbox includes a template and atlases to help with spinal cord MRI (19). However, only few studies, not limited to MS, have used the electro-/magnetospinography, the equivalent of EEG/MEG for the spinal cord. Hence, we believe that SPS neuroimaging is feasible, with the possibility of DIT in illustrating neural connections and disconnections associated with this syndrome and resting-state fMRI in determining which regions have enhanced or decreased connectivity following a symptomatic therapy, such as cannabinoids, in the brain and the spinal cord.

## Treatment of the spasticity-plus syndrome with cannabinoids

Published papers on the SPS consider cannabinoid-based medicines, more specifically nabiximols, as the optimal symptomatic therapy for treating the SPS (2–4). Cannabinoid receptors 1 and 2 are abundant in the CNS, with a higher concentration in the brainstem (2, 3). In MS, voltage-gated channels are reduced, and action potential propagation is compromised (3). Neurons activate compensatory mechanisms such as an ectopic expression of sodium voltage-dependent channels, which can lead to axonal damage in the long-term (3). Preclinical studies showed that cannabinoids reduce the hyperexcitability of sodium voltage-dependent channels and diminish the excitotoxic sodium and calcium currents (3). Hence, cannabinoids can act on these channels to relieve MS symptoms.

Clinical studies previously assessed the efficacy and safety of cannabinoids in the symptomatic treatment of MS. Systematic literature reviews reported benefits, harm, or no significant change from cannabinoids for various parameters such as reducing the self-reported spasticity and pain, no effect on health-related quality of life, and worsening for some cognitive functions (Figure 1) (5, 6). The evidence from these reviews was considered very low to moderate. Conclusions lack robustness, due to different study designs and objectives, and less information on the treatment's dosage, duration, and frequency.

Recently, the response of spasticity-associated symptoms to nabiximols was assessed in an Italian MS population for up to 18 months (20). This retrospective observational study reported 55.6% of the study population (*n* = 1138) who discontinued the treatment, mostly due to lack of effectiveness and adverse events, and 34.9% who maintained it over the total 18-month study period (20). Out of 397 PwMS who continued nabiximols for the overall study period, 363 (91.4%) improved by at least 20% (threshold defining initial responders) and 239 (60.2%) improved by at least 30% (clinically meaningful threshold, defining clinically relevant responders) in their self-reported spasticity, measured by the Numeric Rating Scale (20). At baseline, the most common spasticity-associated symptom was pain, followed by sleep disturbances, spasms/cramps, bladder dysfunction, clonus, mood disorders, and trigeminal neuralgia (20). All symptoms showed a resolution rate of over 48% at 18 months, observed in treatment continuers and for whom data was available (*n* = 179) (20). Results for these associated symptoms were not limited to spasticity responders, which shows that self-reported spasticity is not sufficient in defining PwMS who respond to nabiximols.

## Discussion

A new cluster of spasticity-related symptoms, the SPS, is suggested. A Spanish expert panel attempted to cluster the most common MS symptoms according to their joint onset or a common pathology (4). Both clustering methods can help establish new tools or optimizing the existing questionnaires to better assess the SPS. In fact, the clustering based on a common pathophysiology is more relevant than the one with a joint onset, because it represents a greater potential to find a common therapeutic strategy. Since the SPS includes a vast array of symptoms, it could change the definition of responders to symptomatic therapies. A wider discussion is needed to clearly define this SPS.

Imaging techniques can help illustrate the involved pathways for MS symptoms in the encephalon and the spinal cord. Multiple network connectivity studies in MS exist, particularly for cognition, pain, fatigue, and disability. In their small-sized study, Koenig et al. (21) suggested the Structural and Functional Connectivity Index, a metric tool combining both connectivity methods. A low index over time illustrates declining connectivity and implies disease worsening. They reported a decreasing index over a 2-year study period in PwMS and a positive correlation with behavioral assessment tools, including the MSFC (21). Due to the highly specialized methodology used in this study and its challenging implementation in a larger-sized scale, authors invited future research for simplification (21).

The SPS is a new concept encompassing spasticity and its associated symptoms. While research groups on the SPS considered the cannabinoids as a single potential therapy to relieve the symptoms' burden, the evidence supporting their efficacy is weak. In fact, direct treatment comparisons are needed to determine the optimal treatment strategy for such a syndrome. Additional research is needed to validate this concept and to assess the role of cannabinoids in its symptomatic management. Once established, clinical scales and neuroimaging results should correlate to support this syndrome's validation. Spasticity is also seen in other upper motor neuron diseases such as spinal cord injury, cerebral palsy, and stroke (22). However, the SPS was not generalized for other diseases than MS (2, 3). Research is needed to establish if this syndrome is applicable to diseases other than MS with different weighting for each symptom.

## Author contributions

KAM drafted the manuscript and PD critically reviewed its content. All authors approved the submittedmanuscript.

## Conflict of interest

KAM declared receiving personal fees from Certara outside of this work and a doctoral scholarship from a public grant attributed to the research group by the Canadian Institutes of Health Research and the Canadian Multiple Sclerosis Society (IRSC-MSSC/02088-000). PD disclosed receipt of this public grant. Both authors also declared receiving institutional research support from the Centre de recherche du Centre hospitalier de l'Université de Montréal for projects outside of this publication, including the SENCRL Grant.

## Publisher's note

All claims expressed in this article are solely those of the authors and do not necessarily represent those of their affiliated organizations, or those of the publisher, the editors and the reviewers. Any product that may be evaluated in this article, or claim that may be made by its manufacturer, is not guaranteed or endorsed by the publisher.
